# Agri-food supply chain resilience: An exploration of influencing factors based on fuzzy-DEMATEL-ISM analysis

**DOI:** 10.1371/journal.pone.0338492

**Published:** 2025-12-10

**Authors:** Min Zhang, Jining Yang

**Affiliations:** 1 Office of State-owned Assets, Neijiang Normal University, Neijiang, Sichuan, China; 2 The School of Artificial Intelligence, Neijiang Normal University, Neijiang, Sichuan, China; Hamad Bin Khalifa University College of Science and Engineering, QATAR

## Abstract

Increasingly frequent disruptions from diseases, disasters, and human activities pose a significant challenge to the resilience of the agri-food supply chain (AFSCRE). This study systematically explores the factors influencing AFSCRE and their mechanisms of action by integrating fuzzy Decision-Making Trial and Evaluation Laboratory (DEMATEL) and Interpretative Structural Modeling (ISM). Based on bibliometrics and expert interviews, a three-dimensional indicator system (12 key factors) contains flexibility, agility, and visibility. Triangular fuzzy numbers were used to process expert rating data, and combined with the Converting Fuzzy Numbers into Crisp Scores (CFCS) defuzzification method to establish a total influence matrix. The strength of the influence relationships between factors was determined through the setting of appropriate thresholds, which ultimately results in a five-layer hierarchical structure. The research results show that: level of application of digital technologies, information system maturity, information sharing and synergies, data sharing and analysis capacity and risk management capacity constitute the deep driving factors; degree of simplification of the supply chain structure and level of inventory management are the direct surface factors, presenting isolated characteristics. The study proposes resilience enhancement strategies such as supplier diversification, blockchain traceability technology embedding, and multi-body collaborative decision-making mechanisms, which provide decision support for coping with climate change and public health emergencies.

## Introduction

As the pace of economic integration globally has accelerated and population growth has continued, the position of the agri-food supply chain in the global economic structure has also become more prominent. The agri-food supply chain includes the whole chain of agricultural production, processing, transportation, and storage to marketing, and its efficiency and stability directly translate into international food security, sustainable agricultural development, and the economic interests of the participating industries [1]. However, over the past few years, the agri-food supply chain has faced many unprecedented challenges, leading to significant vulnerabilities and underscoring the critical need for enhanced resilience. The recurring nature of extreme weather events due to climate change, such as droughts, floods, and hurricanes, has not only ruined agricultural production but also severely impacted regional yields and the overall quality of agri-food, leading to substantial economic ramifications for producers and threatening food security [2]. According to the Food and Agriculture Organization (FAO), total agricultural losses from disasters reported in the Sendai Framework Monitor amount to an average of USD 13 billion per year, mostly from floods (16 percent), fire and wildfire (13 percent) and drought (12 percent). Such climate-induced agricultural losses directly translate into significant productivity declines and market volatility. Additionally, global public health events, such as COVID-19, also enhance the exposure of agri-food supply chains [3]. When there is an epidemic, disruptions in logistics, instability of market demand, and shortage of labor occur, and the agri-food supply chain’s regular operation is heavily affected. Meanwhile, the nature of agricultural products’ perishability, seasonality of production, and consumption, as well as the characteristics of supply chain structure with extensive links and decentralization, also render it more susceptible to numerous types of uncertainty [4]. These challenges not only affect the stable supply of agricultural products but also pose a threat to the sustainable development of the agricultural industry, so it has become imperative to improve the resilience of the agri-food supply chain.

The term ‘resilience’ was initially applied to multiple disciplines such as physics, psychology, ecology, and economics [5], and has been widely studied and discussed. Scholars generally describe it as ‘the ability of a system to maintain its function when faced with new disturbances’. Supply chain resilience can be understood as the supply chain’s capability to rapidly recover and continue operations in a usual way despite experiencing diverse unforeseen disruptions and events of uncertainty [6]. In agri-food supply chains, resilience is not only reflected in the ability to cope with external shocks such as natural disasters and public health events, but also in the comprehensive ability of internal management, partnerships, technological innovation, and other aspects [7,8]. However, there are relatively few studies on the resilience of the agri-food supply chain (AFSCRE), most of which are elaborated only at the theoretical level and lack the validation and application of practical cases [9–12], and in the identification of the influencing factors, Coopmans, I., et al. (2023) mentioned three types of resilience capacities: anticipatory, coping, and responsive capacities [13]. Zhao et al. (2024) investigated the AFSCRE capability factors through a cross-country comparative analysis [14]. Zhong et al. (2024) identified 15 factors through literature word frequency, highlighting supply chain leadership and government support [15]. Numerous scholars have discussed and judged the factors influencing the AFSCRE from various aspects and have drawn sufficient conclusions, but there has not been any research on the level of motivational factors.

This study aims to integrate the indicators of flexibility, visibility, and agility; construct a multi-dimensional scoring system; comprehensively assess the framework for evaluating the factors influencing the AFSCRE; identify the key influencing factors and their interrelationships through scientific methods; and provide targeted resilience enhancement strategies for agri-food supply chain managers. While the current research focuses on these operational and structural enablers derived from an extensive analysis of influencing factors, it is acknowledged that a holistic understanding of agri-food supply chain resilience also encompasses critical economic dimensions, such as the financial viability and cost-benefit analyses of resilience strategies [8,10], and complex social dynamics, including stakeholder collaboration mechanisms, particularly within smallholder systems [4,16,17]. These broader aspects, while not the central analytical focus of the approach employed here, form an essential backdrop to the operational resilience explored. The theoretical significance of this study is to enrich and improve the research content in the field of supply chain resilience and to provide new perspectives and methods for the resilience assessment of agri-food supply chains. Constructing a system of influencing factors and an analytical model, can provide a foundation for subsequent empirical research and theory development. The practical significance is to provide decision-making support for agri-food supply chain managers, help them identify key risk points, optimize resource allocation, and formulate effective resilience enhancement strategies to enhance the coping ability of the agri-food supply chain in the face of various uncertainties, guarantee the stable supply of agricultural foods, and promote the sustainable development of the agricultural industry.

To achieve the above research objectives, this study uses the integration of fuzzy-DEMATEL-ISM methodologies to systematically investigate the factors that influence the AFSCRE. A systematic building of the entire system of factors influencing the AFSCRE is constructed through literature investigations, expert interviews, and actual case analysis. Third, fuzzy set theory is applied to manage the uncertainty in the scores of the expert and the DEMATEL approach is used to investigate the causal relationship between factors to determine the importance ranking of the factors. Fourth, ISM method is used to classify the influencing factors hierarchically and construct a multilevel hierarchical model for efficiently presenting hierarchical relationships and paths of transmission among the factors. This integrated approach was specifically chosen for its capacity to elucidate complex causal interdependencies and hierarchical structures among influencing factors, an analytical depth not typically afforded by traditional multi-criteria decision-making (MCDM) models such as AHP or TOPSIS when dealing with systemic factor analysis [18–20].

## Materials and methods

This section will introduce the establishment of the influencer system and a combination of fuzzy-DEMATEL-ISM methods.

### The influence factors system

In this paper, the keywords ‘supply chain resilience of agricultural foods’, ‘supply chain resilience’, and ‘supply chain of agricultural foods’ were used to screen the literature. 268 papers were screened through searches such as the Web of Science, Google Scholar, and CNKI et al. Finally, duplicate and irrelevant literature was removed, and 81 research papers were screened to match the current topic. And the influencing factors mentioned in these studies were meticulously extracted, summarized, and categorized using Microsoft Excel, focusing on those repeatedly highlighted as critical for the comprehensive ability of AFSCRE. This literature-driven process served as the primary basis for identifying a robust set of candidate factors.

Regarding the number of experts needed for fuzzy-DEMATEL, previous successful case studies, such as those by Wang Linxiu et al. (2018), suggest that a range of 5-7 experts can achieve the desired results [21]. In order to validate and refine this literature-derived set of influencing factors, the research team distributed questionnaires to seven experts who are based on their recognized expertise, diverse backgrounds within the agri-food supply chain (academia, industry, technical services, and public sector),and willingness to participate, aiming for a comprehensive and balanced perspective. The seven respondents included two experts with the title of Associate Professor and above, one technician with the title of Intermediate Engineer, one manager of an agricultural cooperative, one employee of an agricultural marketing company, one service provider in the agri-food supply chain, and one civil servant in the agricultural sector, chosen for their diverse and extensive experience across the AFSCRE spectrum.

The validation process involved presenting the preliminary list of factors to the expert panel. Experts were asked to assess the relevance, comprehensiveness, and clarity of each factor in the context of the AFSCRE. They were encouraged to suggest modifications, mergers, or additions to the factor list based on their practical experience and theoretical knowledge. Through this iterative process of expert consultation and consensus-building, the initial set of factors was refined to the final 12 key observational variables presented in Table 1. This expert validation step was crucial for ensuring the content validity and practical applicability of the selected factors, thereby providing an empirical grounding complementary to the literature review. These 12 factors cover three core dimensions: flexibility, agility, and visibility, which are consistently emphasized in resilience literature [22].

**Table 1:** Influencing factors system of the AFSCRE.

Scope	Factors	References
Flexibility [22]	Degree of simplification of the supply chain structure (a1)	[13,23–27]
	Diversity of suppliers (a2)	[9,11,13,26,28]
	Level of inventory management (a3)	[13,16,29–32]
	Capacity to adjust production schedules (a4)	[17,26,28,33–35]
Agility [22]	Information sharing and synergies (a5)	[11,16,17,26,30,33,34,36,37]
	Logistics and distribution efficiency (a6)	[12,17,30,38,39]
	Level of application of digital technologies (a7)	[40–45]
	Organizational and management capacity (a8)	[23,30,46–48]
Visibility [22]	Information system maturity (a9)	[16,40,49]
	Tracing the level of technology application (a10)	[27,30,44,50–53]
	Data sharing and analysis capacity (a11)	[46,49,50,54–56]
	Risk management capacity (a12)	[37,46,57–59]

The selection and prioritization of flexibility, agility, and visibility as the core dimensions for structuring the influencing factors in this study are grounded in extensive bibliometric analysis and expert consensus. These three dimensions are consistently highlighted in foundational and contemporary supply chain resilience literature as fundamental capabilities essential for organizations to effectively anticipate, absorb, adapt to, and recover from disruptions [6,22]. For instance, flexibility underpins the adaptive capacity of the supply chain [22], agility enables rapid response to market changes and disruptions [60], visibility provides the necessary transparency and information flow crucial for proactive and reactive decision-making in resilient systems [61]. While other dimensions such as overarching economic viability or specific policy alignments are critical for the holistic success and sustainability of agri-food supply chains, this study focuses on flexibility, agility, and visibility as they represent the core operational and structural pillars upon which broader resilience strategies and outcomes are often built. They are considered primary enablers that directly influence a supply chain’s intrinsic ability to maintain functionality during and after adverse events.

#### Flexibility.

Agri-food supply chain flexibility has a prominent position in supply chain resilience. Supply chain resilience is the ability of a supply chain to quickly adapt, recover, and keep functioning in the face of internal and external disruptions, and flexibility is a characteristic of the supply chain. When the supply chain is very flexible, it will be able to cope with a variety of disturbances and shocks more efficiently, reduce the disruption time and recovery expense, and increase the stability and competitiveness of the overall supply chain [32]. Flexibility has been regarded by scholars as an important indicator for supply chain resilience, which is utilized to investigate the performance of supply chains in dealing with uncertainty and risk, and is commonly evaluated according to the degree of simplification of the supply chain structure, the diversity of suppliers, the level of inventory management, and the capacity to adjust production schedules. Optimizing the supply chain structure, can reduce the links and complexity, improve the efficiency of information transmission and decision-making, and thus increase the flexibility and responsiveness of the supply chain [27]. Diversity of suppliers can reduce the dependence on a single supplier, and once there is a supplier problem, it can switch to other suppliers in time to maintain supply continuity [28]. Inventory management level, i.e. good inventory management, can reduce the cost of inventory and also inventory risk and meet customer demand. With the optimization of the inventory strategy, for instance, setting safety stock and applying better inventory management technology, the flexibility and responsiveness of the supply chain can be improved [29]. Production plan adjustment ability can regulate the production plan and resource allocation in time according to the fluctuation of market demand to ensure the supply chain’s smooth running and rapid response [30].

#### Agility.

Agility is one of the most powerful means of achieving resilience in agri-food supply chains and is an important component of resilience. Patel and Sambasivan (2022), in a systematic review of the supply chain agility literature, state that agility differs from other similar concepts such as flexibility, leanness, adaptability, and resilience, and is the ability of a supply chain to quickly adjust its network structure and operational strategies to meet customer demand in the face of a dynamic and uncertain market environment. A supply chain can quickly adjust its network structure and operational strategies to adapt to the dynamic demands of customers in the face of dynamic and uncertain market environments [60]. Alfalla-Luque et al. (2023) confirmed a significant positive correlation between supply chain agility and performance through a meta-analysis, which suggests that agility plays an important role in improving the competitiveness and performance of a supply chain [62]. Information sharing and collaboration capabilities are crucial in an agile supply chain. Fernandez-Giordano et al. (2021) stated that one of the antecedents of supply chain agility is the transactional memory system, which facilitates information sharing and knowledge management in the supply chain, thereby improving supply chain agility [63]. Logistics and distribution efficiency is a key component of an agile supply chain. Mishra, Ruchi et al. (2022) in their study emphasized the role of logistics capabilities in achieving supply chain agility, stating that efficient logistics operations increase the responsiveness and flexibility of the supply chain [46]. The degree of application of digital technology has a significant impact on supply chain agility. Fosso Wamba and Akter (2019) explored the impact of supply chain analytical capabilities and agility on supply chain performance in a data-rich environment, stating that the application of digital technology improves supply chain agility and data processing capabilities [64]. Organizational and managerial capabilities are the foundation of supply chain agility. Mahak Sharma et al. (2023), in their study on the fresh food supply chain, highlighted the important role of organizations in improving supply chain agility and sustainability [65].

#### Visibility.

Supply chain visibility refers to the extent to which stakeholders have access to realtime data about the location, status, timing, and accuracy of events occurring throughout the supply chain [66]. This kind of visibility is critical to improving the AFSCRE by increasing the transparency and controllability of the supply chain segments to better cope with uncertainty and risk [61]. According to the 2021-2024 Quadrennial Supply Chain Review, insufficient transparency and data coordination in the agri-food supply chain affects the ability of government and industry to monitor and respond to supply chain risks, which has led to the creation of data platforms and tools by the US Department of Agriculture (USDA) to monitor the functioning of the agri-food supply chain and identify potential supply chain risks [67]. The application of traceability technologies such as IoT, blockchain, and RFID can realize the full traceability of agricultural products from production to consumption, ensuring the accuracy and completeness of product information. This not only helps to improve the transparency and credibility of the supply chain but also ensures product quality and safety by quickly locating and resolving problems as they arise [50]. In the face of risk impact, high-level traceability technology can help enterprises quickly identify the source of the problem and take targeted measures to reduce losses.

### fuzzy-DEMATEL-ISM

**Fig 1:**
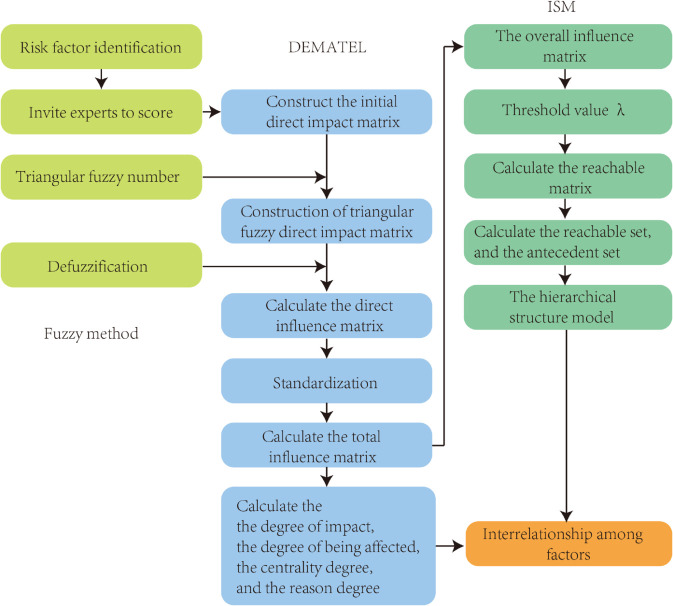
Presents a detailed representation of the fuzzy-DEMATEL-ISM method.

The phases of the proposed fuzzy-DEMATEL-ISM method are presented in Fig 1.

The DEMATEL, a technique often utilized in the analysis and determination of influencing factors, makes full use of the knowledge and insight of experts to deal with a variety of complex social problems and is particularly beneficial in cases where there are ambiguous links between elements [18]. The theory is a new idea developed by Americans to study and solve complex system problems, and a series of studies have been carried out based on graph theory and matrix theory. The DEMATEL method can be used to professionally and systematically analyze the influencing factors among the systems, and the AFSCRE in this paper involves several complex factors. The use of DEMATEL to establish a mathematical modeling method can make the interconnection between the influencing factors concise and quantitative and realize the simplification of the complex system [68]. The DEMATEL method can effectively solve practical problems, especially the complex system of the AFSCRE where the relationship between the factors is not very clear. For the influencing factors of the system, things are bound to each other by a variety of influencing factors in the system, and there are differences in the role of the elements acting inside and outside the system and their complex relationships with each other. Therefore, it is very appropriate to apply the DEMATEL method to its application to the study of the factors influencing the resilience-integrated capacity of the agri-food supply chain. However, the DEMATEL method is based on experts’ knowledge and experience, and the problems of too much subjectivity as well as too many individual differences have a large impact on the research results. Therefore, this study uses the combination of fuzzy Set Theory and DEMATEL to analyze the degree of influence and key factors among the factors influencing the resilience integration capability of agri-food supply chains. Firstly, the subjective scoring of the expert group is quantified using triangular fuzzy numbers, which are integrated into the classical DEMATEL method to weaken the subjective influence of expert scoring to a certain extent [69]. Secondly, the fuzzy numbers are converted into exact values by CFCS (Converting fuzzy Numbers into Crisp Scores) defuzzification [70].

Interpretative Structural Modeling (ISM), first mentioned by Professor J.N. Warfield in 1973 in the United States, is the most commonly used methodology in solving complex and disordered system problems, which are designed in various fields such as social, economic, political, and technological [19]. We use computer-assisted techniques to establish mathematical modeling of the research objectives, identify the role of various influencing factors, and identify the core factors that have a greater impact on the system as a whole. The approach serves as a hierarchy-based perspective to study the key elements and their interconnections within the system as a way to understand the organizational structure and mode of operation within the system. This helps to develop more effective management strategies and decision-making programs, thereby improving the operational efficiency and performance of the system.

The fuzzy-DEMATEL method is able to screen the status and causality of the factors in a complex system, but it cannot clarify its internal structure and logic. ISM technology precisely makes up for this deficiency, and the integration of fuzzy-DEMATEL and ISM methods relatively objectively deals with the dynamic relationship between the factors affecting the AFSCRE, and it can clearly respond to the logical and hierarchical relationships between the factors in the system. Therefore, the fuzzy-DEMATEL-ISM method has obvious advantages in dealing with the dynamic relationship problem of the complex system of agri-food supply chain toughness-influencing factors.

### Model construction

In order to better evaluate the AFSCRE, the three methods of triangular fuzzy number, DEMATEL, and ISM are integrated. In the order of model construction, this paper first applies triangular fuzzy numbers to process the received raw evaluation data and then applies DEMATEL and ISM methods to study the importance and hierarchical structure of factors, respectively.

#### Triangular fuzzy processing.

**Fig 2:**
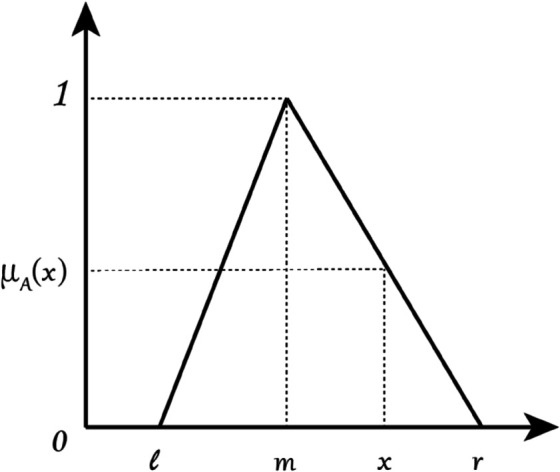
Triangular fuzzy number.

1. Introduction to the triangular fuzzy number method
  A Triangular Fuzzy Number is a simple and intuitive way to represent this kind of uncertain or ‘fuzzy’ information mathematically. They define a range of possibility using three real numbers, typically expressed as A=(l,m,r) where (l≤m≤r). These three real numbers respectively represent the lower bound, the most probable value (or peak), and the upper bound of the triangular fuzzy number.
  The triangle shape is created by a mathematical formula called a membership function, denoted as $\mu_{A}(x)$. It describes the degree of membership for any given value *x*. The function is defined in pieces:
  $$\mu_{A}(x)=\left\{ \begin{array}{c} 0,x<l\\ \frac{x-l}{m-l},l\leq x\leq m\\ \frac{r-x}{r-m},m\leq x\leq r\\ 0,x>r \end{array}\right.,$$ (1)
  The distribution of the triangular fuzzy number is shown in Fig 2.
2. Determination of fuzzy evaluation levels
  The questionnaire designed for expert input requested pairwise comparisons of the identified 12 influencing factors. For each pair of factors (factor *i* and factor *j*), experts were asked to rate the degree of influence of factor *i* on factor *j*. Instead of a traditional Likert scale, experts were guided to provide their judgments using a linguistic scale corresponding to predefined levels of influence. The level of influence between each of the two factors is categorized into five levels, where levels 0, 1, 2, 3, and 4 indicate ‘no influence’, ‘low influence’, ‘medium influence’, ‘high influence’, and ‘very high influence’. A triangular fuzzy number was then assigned to each degree as shown in Table 2. For example, when one expert thought one factor had a medium impact on another, the corresponding triangular fuzzy number was (0.25, 0.5, 0.75). In particular, when all seven experts considered the relationship between the factors to be ‘no influence’, there was no influence between them, and the triangular fuzzy number was no longer calculated. This approach allows for the inherent vagueness and subjectivity in expert opinions to be captured more effectively than discrete numerical scales [69]. The survey instrument clearly defined each factor and provided instructions on the pairwise comparison task to ensure clarity and consistency in responses.
  Firstly, all the scores of the experts are converted into triangular fuzzy numbers in the form of $\left(l_{ij}^{k},m_{ij}^{k},r_{ij}^{k}\right)$, which represents the degree of influence of the factor *i* on the factor *j* according to the expert *k*. Next, the CFCS (Converting the fuzzy data into Crisp Scores) processing method is applied to denazify the fuzzy numbers. The specific calculation steps are as follows:
  i. Normalization
    The triangular fuzzy number of each expert’s score was standardized by Eqs (2) to (4).
    $$xl_{ij}^{k}=(l_{ij}^{k}-minl_{ij}^{k})/(\Delta_{min}^{max})$$ (2)
    $$xm_{ij}^{k}=(m_{ij}^{k}-minl_{ij}^{k})/(\Delta_{min}^{max})$$ (3)
    $$xr_{ij}^{k}=(r_{ij}^{k}-minl_{ij}^{k})/(\Delta_{min}^{max})$$ (4)
    where, $\Delta_{min}^{max}=\max r_{ij}^{k}-\min l_{ij}^{k}$.
  ii. Calculation of left and right standard values
    The normalized triangular fuzzy numbers are transformed into left and right standard values, respectively, by Eqs (5) and (6).
    $$xls_{ij}^{k}=(xm_{ij}^{k})/(1+xm_{ij}^{k}-xl_{ij}^{k})$$ (5)
    $$xrs_{ij}^{k}=(xr_{ij}^{k})/(1+xr_{ij}^{k}-xm_{ij}^{k})$$ (6)
  iii. Calculation of total standard value
    Calculation of total standard value by Eq (7).
    $$x_{ij}^{k}=\frac{xls_{ij}^{k}(1-xls_{ij}^{k})+xrs_{ij}^{k}xrs_{ij}^{k}}{1-xls_{ij}^{k}+xrs_{ij}^{k}}$$ (7)
  iv. Calculate the clarity value
    Calculate the expert *k* ’s score for the degree of influence of factor *i* on factor *j* by using Eq (8).
    $$a_{ij}^{k}=\min l_{ij}^{k}+x_{ij}^{k}\Delta_{min}^{max}$$ (8)
  v. Calculate the average score
    Eq (9) calculates the average of all experts’ scores on the degree of influence of factor *i* on factor *j*.
    $$a_{ij}^{k}=\frac{1}{k}\sum_{k=1}^{k}a_{ij}^{k}$$ (9)

**Table 2:** The triangular fuzzy numbers correspond to the expert’s judgment.

Value	Level of influence	fuzzy number
0	no influence	(0, 0, 0.25)
1	low influence	(0, 0.25, 0.5)
2	medium influence	(0.25, 0.5, 0.75)
3	high influence	(0.5, 0.75, 1)
4	very high influence	(0.75, 1, 1)

#### Importance analysis of factors based on DEMATEL.

1. Constructing the direct influence matrix
  Obtain the n×n direct influence matrix **A**. The mentioned fuzzy method calculates $𝐀(𝐀={\left[a_{ij}\right]}_{n\times n})$, *a*_*ij*_ denotes the final impact of factor *i* on factor *j*, *a*_*ij*_ = 0 for i=j,(i,j=1,2,3,...,n).
2. Normalization of the direct influence matrix
  Establish the normalized direct influence matrix $𝐁(𝐁={\left[b_{ij}\right]}_{n\times n},0<b_{ij}<1)$.
  $$𝐁=\frac{1}{\max_{1\leq i\leq n}\sum_{j=1}^{n}a_{ij}}𝐀$$ (10)
3. Construction of the total influence matrix
  The total influence matrix $𝐓(𝐓={\left[t_{ij}\right]}_{n\times n})$ is calculated as follows, where **I** denotes the unit matrix.
  $$𝐓=𝐁{\left(𝐈-𝐁\right)}^{-1}$$ (11)
4. Calculation of degree of impact, degree of being affected, the centrality degree, reason degree
  Calculate the degree of impact *D*_*i*_, the degree of being affected *E*_*i*_, the centrality degree *C*_*i*_, and the reason degree *R*_*i*_. The *D*_*i*_ and *E*_*i*_ denote the row and column sums of the matrix **T**, respectively, which can be calculated by Eqs (12) and (13). Next, *D*_*i*_ and *E*_*i*_ are added and subtracted to obtain *C*_*i*_ and *R*_*i*_, as shown in Eqs (14) and (15). *C*_*i*_ represents the degree of importance of factor *i* among all factors, and the greater the centrality degree, the higher the importance of the factor. The reason degree *R*_*i*_ represents the degree of a causal relationship between factor *i* and other factors. If *R*_*i*_ is negative, it means that the factor is greatly influenced by other factors and is called an effect factor. If *R*_*i*_ is positive, it means that the factor has a significant influence on other factors and is called the cause factor.
  $$D_{i}=\sum_{i=1}^{n}t_{ij}(t=1,2,3,...,n)$$ (12)
  $$E_{i}=\sum_{j=1}^{n}t_{ij}(t=1,2,3,...,n)$$ (13)
  $$C_{i}=D_{i}+E_{i}(t=1,2,3,...,n)$$ (14)
  $$R_{i}=D_{i}-E_{i}(t=1,2,3,...,n)$$ (15)
  Since the total influence matrix constructed in the DEMATEL method does not consider the role of each factor in influencing itself, to consider this factor in the overall influence matrix, this paper uses the unit matrix **I** to reflect the correspondence of the factors themselves. The specific approach is to add the total influence matrix **T** and the unit matrix **I** through Eq (16) to get the overall influence matrix **H**.
  𝐇=𝐓+𝐈 (16)

#### ISM-based factor hierarchy model construction.

1. Generate the adjacency matrix
  Generate the adjacency matrix **K** through the overall influence matrix **H**. Firstly, determine the threshold value *λ*, which determines the complexity of the structure. Different values of *λ* correspond to different reachable matrices, which will get different hierarchical structure models. When *λ* is large, the hierarchical structure of factors is more complex, and when *λ* is small, the hierarchical structure of factors is simpler. In the calculation process, it should be determined according to the specific situation of the research object. There are three ways to determine the threshold *λ* as follows:
  - Determine the threshold *λ* based on the experience of experts. This threshold determination method is usually based on the expert’s judgment of the complexity of the hierarchical structure model.
  - Take the sum of the mean and standard deviation of the factors of comprehensive influence as the threshold *λ*.
  - The optimal threshold *λ* is obtained by substituting numerical trial calculations. Alternative threshold *λ* is initially determined empirically, and *λ* is determined by substituting calculations to obtain the node degree of the factors. The node degree, representing the total number of incoming and outgoing links for a factor, serves as an indicator of its connectivity and influence within the system structure at a given *λ*. In the context of network analysis and ISM, the ’node degree’ (or simply degree) of a factor (node) in the system is a measure of its connectivity. For a directed graph represented by the reachable matrix **M**, the node degree for a factor *i* can be defined as the sum of its out-degree (number of factors it influences, i.e., sum of row *i*) and its in-degree (number of factors that influence it, i.e., sum of column *i*). A higher node degree generally indicates a more influential or more affected factor within the network structure established by the reachable matrix [71]. Where the sum of the elements of row *m*_*i*_ (out-degree) and column *m*_*j*_ (in-degree) corresponding to a factor in the matrix M is considered as the node degree of the factor (degree_*i*_ = out-degree_*i*_ + in-degree_*i*_). After the threshold *λ* is determined, the elements in the overall system influence matrix are converted to 0 or 1 according to Eq (17) to generate the adjacency matrix **K**.
    $$k_{ij}=\left\{ \begin{array}{c} 1,k_{ij}\geq \lambda \\ 0,k_{ij}<\lambda \end{array}\right.$$ (17)
2. Establish the reachable matrix **M**.
  The reachable matrix **M** is a fundamental concept in Interpretative Structural Modeling (ISM), representing the direct and indirect relationships between the elements (factors) in a system [19]. It is a binary matrix where an entry *m*_*ij*_ = 1 indicates that factor *j* is reachable from factor *i* through some path (either direct or indirect), and *m*_*ij*_ = 0 indicates that factor *j* is not reachable from factor *i*. The reachable matrix thus shows whether one factor can influence another, considering transitive relationships within the system. The reachable matrix **M** is obtained using the Boolean operation of the adjacency matrix **K**. The formula is as follows:
  $${𝐊}_{\;}^{i-1}\neq {𝐊}_{\;}^{i}={𝐊}_{\;}^{i+1}(i=2,3,4,...,n)$$ (18)
  This iterative process ensures that all indirect links are captured, transforming the initial adjacency matrix **K** (representing direct relationships) into the comprehensive reachable matrix **M**.
3. Calculate the levels of all factors and model the hierarchy of the system.
  The reachable matrix **M** utilized a reachable set *P*, a antecedent set *Q*, and a common set *O*, where O=P∩Q. The reachable set is the set of factors corresponding to the columns in which all the elements with 1 in row *i* of the reachable matrix **M** are located, representing all the possible arrivals of factors *m*_*i*_ ; the antecedent set is the set of factors corresponding to the rows in which all the elements with 1 in column *i* of the reachable matrix **M** are located, representing all the possible arrivals of factors at *m*_*i*_.
4. Draw a multi-level hierarchy.
  Based on the relationship between the elements and the extraction results of the hierarchical structure, by integrating the influence values between the variables in the influence matrix, as well as by using the ranking results of centrality and reason, it is possible to draw a hierarchical structure with integrated influence values. The reachable relationships between the influencing factors are represented by directed line segments. The upper layer means that the influencing factors are direct, and the lower layer means that the influencing factors are rooted.

### Ethical considerations

Neijiang Normal University has no institutional review board for social-science research. The study employed an anonymous online questionnaire directed at supply-chain experts; no sensitive personal data were collected and risk was no more than minimal. Procedures followed the ICC/ESOMAR International Code on Market and Social Research (2022) and the Helsinki Declaration as applied to anonymised social-science surveys. Voluntary electronic informed consent was obtained from every participant before the questionnaire could be accessed; respondents could withdraw at any time by simply closing the browser. No monetary or non-monetary incentives were provided.

## Results

Table 1 lists the 12 factors that influence the AFSCRE. 7 experts and scholars with project-related work experience were invited to score the factors. The scoring results show the influence of one factor on other factors and the final influence value *a*_*ij*_, which is used to form a direct influence matrix **A** according to Eqs (2)–(9). And then matrix **A** is normalized to Matrix **B** according to Eq (10). Matrix **T** is calculated by Eq (11). After obtaining the matrix **T**, Eqs (12)–(15) calculate $D_{i},E_{i},C_{i},R_{i}$. While the supplementary material provide detailed numerical data from the DEMATEL analysis.

**Fig 3:**
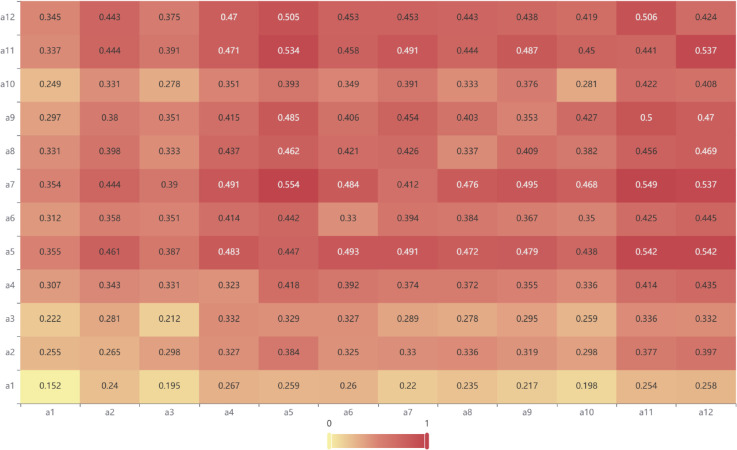
Heatmap of the total influence matrix for AFSCRE factors.

To better illustrate the findings, the total influence matrix and the DEMATEL indicator values are further visualized. Fig 3 (Heatmap of the Total Influence Matrix) presents the total influence matrix **T** as a heatmap. In this visualization, rows represent the influencing factors (a1 - a12) and columns represent the influenced factors (a1 - a12). The intensity of the color in each cell directly corresponds to the strength of the influence *t*_*ij*_ exerted by the row factor on the column factor, with warmer/darker colors (e.g., dark red) indicating stronger influences and cooler/lighter colors (e.g., light yellow) indicating weaker ones. This heatmap allows for a rapid visual assessment of the most significant direct and indirect influence paths with in the system, highlighting which factors are strong influencers or recipients of influence.

**Fig 4:**
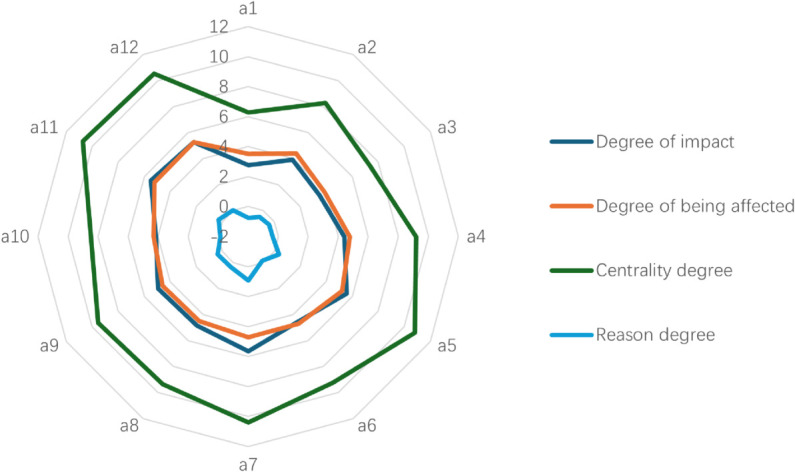
DEMATEL Metrics Radar Chart for Key AFSCRE Influencing Factors.

To further elucidate the characteristics of the most dominant factors, Fig 4 (DEMATEL Metrics Radar Chart) displays the degree of impact(D), degree of being affected(E), centrality degree(C), and reason degree (R) for selected key influencing factors: a5, a7, a9, a11 and a12. Each axis of the radar chart corresponds to one of these four DEMATEL metrics, and each factor is represented by a distinct colored polygon. The shape and extent of each polygon visually articulate the factor’s profile.

**Fig 5:**
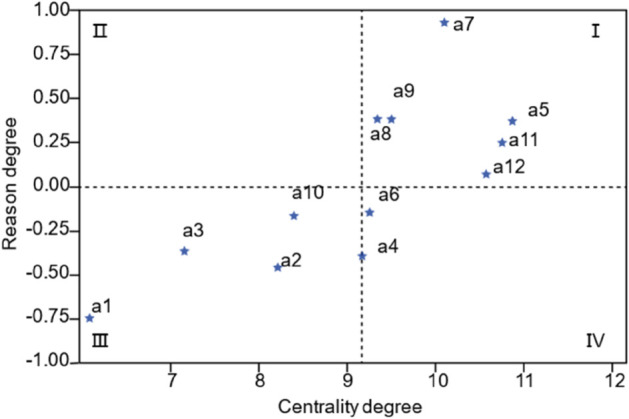
Centrality degree and reason degree distribution diagram.

The centrality degree and the reason degree distribution diagram are presented in Fig 5 to display the distribution of each element more intuitively. In Fig 5, a5, a7, a8, a9, a11, and a12 are located in the first quadrant, indicating that they have a very significant impact on the formation of the research object and a substantial influence on other outcome-oriented factors. a1, a2, a3, and a10 are located in the third quadrant, indicating that these factors are the result of the combined effect of other causal factors and have a certain impact on the formation of the research object. Other factors are also the result of the combined effect of other causal factors, but they have a very important impact on the formation of the research object.

The key issue in constructing the matrix **M** is to choose the threshold *λ*. This threshold determines which influence relationships are deemed significant for structuring the model. To ensure the robustness and reliability of the derived hierarchical model, and to understand the impact of *λ* on model complexity, a sensitivity analysis was conducted. This involved setting *λ* at ten distinct values within the range of 0.41 to 0.50, based on the principles outlined in the methodology (statistical properties of the influence matrix, iterative testing, and expert validation of model interpretability).

**Fig 6:**
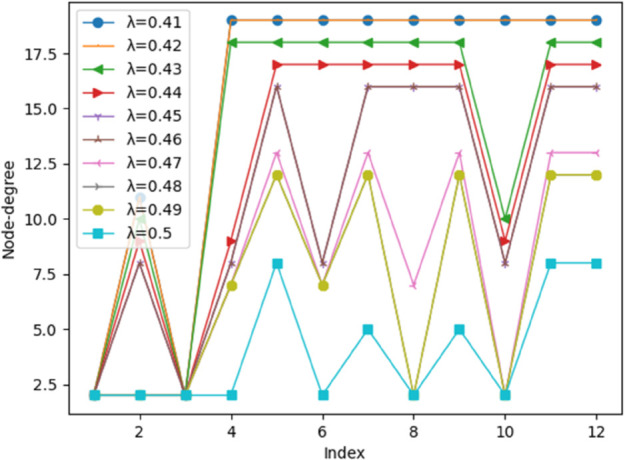
Scatterplot of node degree for factors at different thresholds.

For each of these ten *λ* values, an adjacency matrix **K** was generated, followed by the corresponding reachable matrix **M**. The hierarchical complexity, as indicated by the ’node degree’ of each factor, was then analyzed across these different thresholds. Fig 6 presents a scatterplot illustrating the node degree for each of the 12 factors across these ten different *λ* thresholds. This node degree helps in understanding the overall connectedness and potential structural importance of each factor under varying threshold conditions for establishing links in the adjacency matrix **K**, which subsequently forms the basis for the reachable matrix **M**.

**Fig 7:**
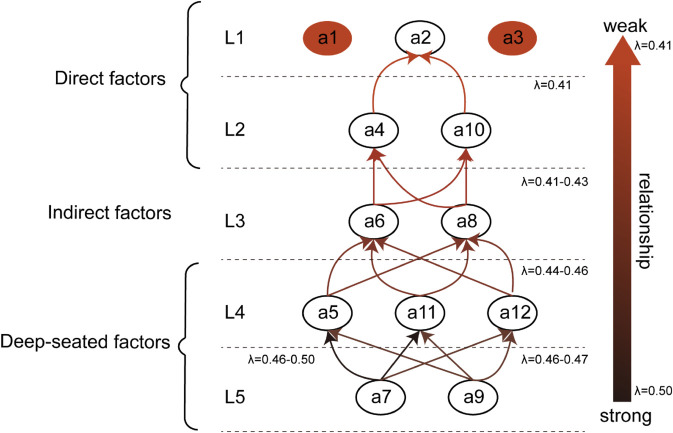
Relationship diagram among factors. The five-level hierarchical structure of this model was established using the ISM method. Arrows indicate the direction of influence. The strength of influence, filtered based on thresholds, is represented by connecting lines: darker lines denote stronger relationships (λ≥0.45), while lighter lines indicate weaker relationships (0.41≤λ<0.45). It should be specifically noted that factors a1 and a3 appear as isolated nodes, as their interactions with other factors did not reach the significance threshold, highlighting their context-dependent nature. The vertically arranged structure illustrates the propagation path of influence from deep-level driving factors (lower tier) to direct surface-level factors (upper tier).

This visual representation in Fig 6 effectively serves as a sensitivity analysis, demonstrating how the connectivity and structural prominence (node degree) of individual factors, and thus the overall model complexity, vary with changes in *λ*. This figure reveals a key phenomenon: although the degree of certain factors (i.e., the number of connections) fluctuates with adjustments to the *λ* value, this primarily reflects the inclusion or exclusion of weaker, borderline influence pathways within the system. However, the most significant finding of this analysis is that the model’s five-level hierarchical structure exhibits high stability across the entire tested range of *λ* values (0.41 to 0.50). This stability strongly demonstrates that the core, high-intensity causal relationships forming the system’s backbone are sufficiently robust to remain unaffected by threshold fine-tuning. Specifically, while factors like a6 (logistics distribution efficiency) and a8 (organizational management capability) experienced fluctuations in their connection counts, their central roles as intermediary transmission factors remained unchanged. Similarly, deep-level drivers (e.g., a7, a12) consistently remained anchored at the model’s base, while direct surface factors (e.g., a1, a3) persistently occupied the top layer. Thus, this sensitivity analysis confirms that the constructed hierarchy is robust and not an artifact of specific threshold selections, providing strong support for the model’s reliability. The consistent emergence of a meaningful five-layer structure within this *λ* range, despite these minor variations in node connectivity, lends strong confidence to the reliability of the overall hierarchical model presented. And the hierarchical structure model (Fig 7) is then established based on an optimally chosen *λ* from this tested range that best satisfies these criteria.

The locations of the system factors are selected sequentially in the figure, and the interconnections between the factors are labeled. As presented in Fig 7, twelve factors are divided into five layers, and the arrow direction indicates the mutual influence relationship between two events. For example, the basic event a7 with an arrow pointing to a5, indicates that a7 affects a5. The factors at the lower layers are internal reasons. Meanwhile, factors at the higher layers indicate a more direct impact on the AFSCRE. To distinguish the strength of the interrelationships between factors more intuitively, the interrelationships between factors in the model structure are color-coded, with darker colors representing stronger interrelationships between factors.

Furthermore, factors are categorized into direct, indirect, and deep-seated factors based on the influence of the factors. The top two layers in the model are direct factors of the AFSCRE. The middle layer is the indirect factors, which generally do not directly affect resilience but can influence direct factors. The bottom two layers are deep-seated factors that may not directly affect resilience, but long-term adverse conditions can affect direct or indirect factors. In Fig 7, a5, a7, a9, a11, and a12 with higher reason degrees in Fig 5 appear in the lower layers as the deep-seated factors. a1, a2, a3, and a10 with lower reason degrees appear in the higher levels as the direct factors.

## Discussion

While previous studies, such as the cross-national comparisons by Zhao et al. (2024) and the 15-factor model by Zhong et al. (2024), have successfully identified key factors influencing AFSCRE, this study offers a distinct theoretical advancement through its methodological integration [14,15]. The fuzzy DEMATEL-ISM approach moves beyond mere factor identification to reveal the complex, multi-layered causal hierarchy and dynamic interdependencies within the system, a depth of analysis not typically afforded by non-hierarchical or purely comparative models. Specifically, our hierarchical model uniquely elucidates the causal pathways from deep-seated drivers (e.g., level of application of digital technologies (a7) and risk management capacity (a12)) to the more immediate, surface-level factors. Furthermore, a key novel insight is the identification of the context-dependent nature of isolated factors (a1/a3), whose strategic importance is not determined by systemic influence but by the specific disruption scenario, a nuance that prior factor-listing models do not capture. This demonstrates how the integrated method can uncover not just what the factors are, but how they interact and propagate influence through the supply chain structure.

Based on this hierarchical structure, the first level is: degree of simplification of the supply chain structure (a1), diversity of suppliers (a2), level of inventory management (a3), and the second level is: capacity to adjust production schedules (a4), and tracing the level of technology application (a10), are direct factors, indicating that these factors are the most immediate cause of supply chain disruptions. a1 and a3 in the topology diagram are isolated factors. From the topological map, it can be seen that no directed line segments are pointing from other factors to a1 and a3, and no directed line segments emanating from a1 and a3 pointing to other factors, i.e., the two factors do not have an influence-affect relationship on other factors. Examined from the perspective of the combined influence matrix **T**, the rows and columns corresponding to a1 & a3 have values less than the value of λ(λ=0.41). From the above diagram, it can be seen that the interaction between a1 and a3 and other factors of the system is the weakest, and the influencing effect is the smallest of the two factors.

Relating the isolated elements, degree of simplification of the supply chain structure (a1) and level of inventory management (a3) can be contextualized with real-world resilience needs. For example, in post-disaster recovery scenarios, a simplified supply chain (a1), such as those seen in short food supply chains that reduce intermediaries [23], can expedite the delivery of essential food aid by minimizing logistical hurdles. This aligns with observations during the COVID-19 pandemic where complex, lengthy supply chains faced significant disruptions [2,3]. Similarly, strategically managed inventory levels (a3) become crucial. While large inventories can buffer against short-term supply shocks, as might be pursued following environmental shocks [32], they also carry risks of spoilage and capital tie-up. Conversely, excessively lean inventories in a simplified chain can increase vulnerability if not carefully planned. Thus, the ‘isolated’ nature of these factors suggests they are crucial initial points of adaptation whose optimal configuration depends heavily on the specific context and potential disruptions, rather than being universally driven by other internal system dynamics. Effective inventory strategies in these scenarios allow for quicker adaptation, though the optimal approach must consider the specific nature of the disruption and local capacities to avoid new vulnerabilities such as stockouts or spoilage.

Diversity of suppliers (a2), capacity to adjust production schedules (a4) have a low degree of influence and a high degree of being influenced, reflecting the fact that this factor has less influence and is more susceptible to the influence of other factors. Tracing the level of technology application (a10) is more isolated when comparing the values of centrality, and influence. So some measures should be implemented to ensure the diversity of suppliers, such as a diversified supplier layout, expanding domestic and international supplier networks, establishing regional supply chains through cooperation mechanisms such as the ‘Belt and Road’, and promoting the diversification of import sources; supporting overseas investment by agricultural enterprises, cultivating overseas production bases through arable land leasing and technological cooperation, and forming transnational supply networks. These strategies directly enhance resilience by reducing dependence on single sources or rigid production plans, which is critical when direct factors like supply chain structure (a1) or inventory (a3) are impacted by a crisis.

Next, logistics and distribution efficiency (a6), and organizational and management capacity (a8) are ranked in the middle of the degree of centrality and influence and are ranked in the middle of the ISM model diagram and are able to connect most of the factors. These indirect factors act as crucial enablers, thus, resilience strategies should focus on strengthening the underlying capabilities and infrastructure that support the effective execution of direct, operational responses [13]. Improving logistics and distribution efficiency (a6), which reflects the logistics system’s efficiency and flexibility and directly impacts resilience post-disruption, requires strategies aimed at building robust and flexible logistics networks. This could involve exploring alternative transportation modes and fostering partnerships, thereby enhancing the system’s ability to cope with disruptions [12,38,39,46]. Similarly, bolstering organizational and management capacity (a8), which reflects the effectiveness of decision-making mechanisms and management responses during unexpected incidents, involves developing agile decision-making processes, clear communication protocols, and robust coordination mechanisms to ensure effective crisis management [23,46,48,65]. Notably, in the context of smallholder-dominated systems prevalent in many developing countries, enhancing organizational capacity (a8) requires a deep understanding of social capital. As highlighted by Cimino et al.(2024) and Ali et al.(2023), effective collaboration is not merely a formal structural arrangement but is founded on trust, shared norms, and collective action [17]. Here, information and communication technology (ICT) platforms play a transformative role [16]. As discussed in, these platforms can significantly enhance smallholder resilience by facilitating the information sharing and transparent communication necessary to build and sustain this crucial social capital, thereby strengthening the collective organizational capacity to respond to disruptions.

Finally, information sharing and synergies (a5), data sharing and analysis capacity (a11), risk management capacity (a12), level of application of digital technologies (a7) and information system maturity (a9) are deep-seated factors. At the same time, the above five factors are ranked in the top five among the 12 influencing factors, which indicates that they are closely linked with other factors in the whole system and are in the position of important nodes. These five factors belong to the agility and visibility dimensions, respectively, which indicates that in the process of measuring the AFSCRE, the indicators of agility and visibility can more comprehensively and objectively assess the comprehensive risk-resistant capability of the agri-food supply chain. These deep-seated factors are foundational to long-term resilience, and strategies addressing them require a strategic, systemic approach, often involving significant investments to build anticipatory, adaptive, and transformative capabilities [10,13,34,42].

Information sharing and synergies (a5) and level of application of digital technologies (a7) are ranked second and first in the degree of influence, respectively, indicating that the degree of influence of these two factors on other influencing factors is very large. The degree of being influenced ranked third and fifth, respectively, indicating that these two factors are susceptible to the influence of other factors. Strategies here involve developing integrated digital platforms and fostering collaborative data-driven ecosystems [11,36,37,40,64]. The level of application of digital technologies (a7) is pivotal, with strategies focused on embedding innovations such as IoT and blockchain to enhance transparency, traceability, and overall system intelligence [2,41,43–45,50–53,72]. However, the implementation of these strategies must be contextualized to address regional disparities, particularly the digital divide in developing countries. A one-size-fits-all approach to enhancing the level of application of digital technologies (a7) is impractical. For instance, while full blockchain integration may be a viable goal in technologically advanced regions, a more adaptive and inclusive strategy for many developing nations would be to prioritize low-cost, mobile-based traceability systems. Such systems can leverage existing cellular infrastructure to significantly improve supply chain visibility and accountability without the prohibitive costs and technical barriers associated with blockchain, thereby offering a pragmatic pathway to bridge the digital divide and enhance resilience.

Information system maturity (a9), data sharing and analysis capacity (a11), risk management capacity (a12) are ranked fifth, third, and fourth in terms of the degree of being influenced, indicating that these three factors are most likely to influence other factors and are key nodes that play a decisive role in the comprehensive capacity measurement of resilience in agri-food supply chains. Enhancing these requires improving analytical capabilities for predictive insights [46,49,54–56], strengthening information systems [16,40,49], and establishing proactive risk governance frameworks. This includes embedding risk assessment into strategic planning and cultivating a resilient organizational culture that can anticipate and respond to diverse threats effectively [8,37,46,57,59]. Addressing these deep-seated factors by investing in such systemic capabilities is fundamental to cultivating the overall flexibility, agility, and visibility crucial for comprehensive AFSCRE.

## Conclusion

This study presents a method for calculating the comprehensive risk-resistant capability of the agri-food supply chain based on the fuzzy-DEMATEL-ISM method, providing an approach to analyze the AFSCRE evolution and interactions among factors. The major conclusions are as follows.

First, an indicator system is established for the AFSCRE, including 12 general factors comprising the degree of simplification of the supply chain structure, diversity of suppliers and level of inventory management. Besides, the relationship between every two factors is determined using the fuzzy method.

Second, the DEMATEL method was used to obtain the degree of impact, the degree of being affected, the degree of centrality, and the reason degree. It indicates that information sharing and synergies (a5), data sharing and analysis capacity (a11), risk management capacity (a12), level of application of digital technologies (a7), and information system maturity (a9) are the main factors of the AFSCRE.

Third, the analysis model was developed by the fuzzy-DEMATEL-ISM method. It revealed the hierarchical structure of factors and the evolution of the AFSCRE. It indicates that information sharing and synergies (a5), data sharing and analysis capacity (a11), risk management capacity (a12), level of application of digital technologies (a7), and information system maturity (a9), are deep-seated factors. Some advice is given according to the analysis results. These works can provide a reference list for scholars and practitioners in relevant fields.

The limitations of this paper include the specific scope defined by the fuzzy-DEMATEL-ISM methodology, the number of expert interviews, and certain inherent model assumptions. Firstly, the expert inputs, which form the basis for the initial influence matrix, are largely static, capturing a snapshot of expert perceptions at a specific point in time. The model does not currently incorporate mechanisms for real-time data integration or dynamic updating of these expert judgments, which could be beneficial in a rapidly evolving risk landscape. Secondly, while fuzzy logic addresses the vagueness in expert opinions, the structural relationships derived through ISM, once established, are also relatively static unless the entire analysis is repeated.

In addition, future research could focus on distinguishing between resilience and accessibility by developing a more comprehensive and integrated assessment framework that covers both aspects. Building on the insights and addressing the limitations of the current study, future research directions could also valuably explore:

- The development and application of robust economic resilience metrics, including detailed cost benefit analyses for various resilience-enhancing strategies such as diversification or technology adoption, to better inform investment decisions.
- Amorenuanced exploration of social dynamics within agri-food supply chains, focusing on mechanisms for enhancing stakeholder collaboration, trust, and social capital, particularly in empowering smallholder systems and ensuring equitable resilience outcomes.
- The interplay between policy alignment at various governance levels and its impact on fostering these economic and social dimensions of resilience.
- The development of dynamic extensions to the current fuzzy-DEMATEL-ISM framework. This could involve integrating real-time data streams, potentially from Internet of Things (IoT) devices deployed across the agri-food supply chain, to dynamically update the influence scores or even the structural model itself. For instance, data on logistics disruptions, inventory levels, or environmental conditions captured via IoT could potentially modulate expert-defined weights or trigger re-evaluations of critical factor interdependencies.

These future endeavors could further enhance the practical applicability and predictive power of models aimed at understanding and strengthening agri-food supply chain resilience.

## Supporting information

S1 Data Data analysis results. (XLSX)
